# Optimizing the use of nirmatrelvir/ritonavir in solid organ transplant recipients with COVID-19: A review of immunosuppressant adjustment strategies

**DOI:** 10.3389/fimmu.2023.1150341

**Published:** 2023-04-04

**Authors:** Yangming Tang, Yue Li, Turun Song

**Affiliations:** Department of Urology, West China Hospital, Sichuan University, Chengdu, China

**Keywords:** COVID-19/SARS-CoV-2, drug interaction, immunosuppressant, nirmatrelvir and ritonavir/Paxlovid, pharmacology, solid organ transplantation

## Abstract

The coronavirus disease 2019 (COVID-19) pandemic has caused a significant burden of morbidity and mortality worldwide, with solid organ transplant recipients (SOTRs) being particularly vulnerable. Nirmatrelvir and ritonavir have demonstrated the potential for reducing the risk of hospitalization and death in patients with mild-to-moderate COVID-19. However, ritonavir has a strong drug–drug interaction with CYP3A-dependent drugs such as calcineurin inhibitors, potentially leading to rapid increases in blood concentration. As SOTRs are commonly prescribed immunosuppressants, co-administration with nirmatrelvir/ritonavir requires careful consideration. To address this issue, we conducted a literature review to evaluate the use and adverse effects of nirmatrelvir/ritonavir in SOTRs and explore feasible immunosuppressant adjustment regimens. Our findings suggest that nirmatrelvir/ritonavir could be a feasible treatment option for COVID-19 in SOTRs, provided that appropriate immunosuppressive drug management is in place during co-administration. Although prescribing the novel anti-SARS-CoV-2 drug to transplant recipients poses challenges, potential strategies to overcome these issues are discussed. Further studies are needed to determine the optimal dosing strategies of nirmatrelvir/ritonavir, immunosuppressant adjustment, and monitoring in this patient population.

## Introduction

The coronavirus disease 2019 (COVID-19) pandemic has persisted for nearly 3 years, resulting in significant morbidity and mortality worldwide (1). Despite global efforts, combating severe acute respiratory syndrome coronavirus 2 (SARS-CoV-2) has remained challenging. The emergence of the Omicron variant in November 2021 has further complicated the situation, leading to rapid global spread and becoming the predominant variant in circulation (2). Recent *in vitro* studies have shown that many therapeutic monoclonal antibodies have lower neutralizing activity against the Omicron variant compared to earlier strains (3). As a result, there is an urgent need for the development of effective drugs to treat COVID-19.

Paxlovid™ (Pfizer, New York City, NY, USA) is a novel oral antiviral drug from Pfizer that contains nirmatrelvir and ritonavir (NR). In a phase II–III clinical trial, NR demonstrated an 89% reduction in the risk of hospitalization or death within 28 days compared to a placebo in 2,246 unvaccinated patients with mild COVID-19 (4). On 22 December 2021, the US Food and Drug Administration (FDA) approved an emergency use authorization (EUA) for Paxlovid, approving it for outpatient treatment of mild-to-moderate COVID-19 patients who are at risk for progression (≥ 12 years of age and weighing ≥ 40 kg) within 5 days of symptom onset (5). Since then, NR has emerged as a promising treatment option for patients infected with SARS-CoV-2 during the Omicron surge, demonstrating inspiring results against the Omicron variant (6).

Nirmatrelvir is an oral protease inhibitor with *in vitro* pan-human coronavirus antiviral activity. It targets M^PRO^, a viral protease that plays a crucial role in viral replication and exhibits excellent off-target selectivity and *in vivo* safety profiles (7). Ritonavir is another component of NR and acts as an HIV-1 protease inhibitor and a cytochrome P450 3A (CYP3A) inhibitor, which is necessary to achieve adequate concentrations of nirmatrelvir. However, the interaction between ritonavir and CYP3A-dependent drugs can result in a rapid and substantial increase in blood concentrations of the latter drugs (8).

Immunosuppressive drugs (ISDs) such as calcineurin inhibitors (CNIs) (e.g., tacrolimus and cyclosporine) and mammalian target of rapamycin (mTOR) inhibitors rely heavily on CYP3A metabolism (9), which can lead to drug interactions and potentially harmful increases in blood concentrations if co-administered with NR. It is therefore recommended to adjust the dose of the ISDs and monitor concentrations and immunosuppressant-associated adverse reactions if co-administered with NR (10). Patients with transplants are particularly vulnerable to COVID-19 morbidity and mortality due to immunosuppression, comorbidities, and suboptimal response to vaccination (11). Given the current surge of SARS-CoV-2 and the Omicron variant, it is likely that transplanted patients will be prescribed Paxlovid more frequently. However, there is currently a paucity of clinical data available regarding the use of Paxlovid in solid organ transplant recipients (SOTRs), and no consensus exists regarding the adjustment of CNIs used in these patients. Therefore, there is an urgent need to gather available data on this subject. In this paper, we conducted a literature review to summarize the adverse reactions associated with Paxlovid administration in SOTRs and explore feasible protocols for adjusting ISDs dosages.

## Materials and methods

We conducted a comprehensive literature search from 1 December 2021 to 15 November 2022, using the Medline and Embase databases. The search strategy included the following keywords: Paxlovid, nirmatrelvir or PF-07321332, ritonavir, COVID-19, solid organ transplant (SOT), kidney transplantation, liver transplantation, heart transplantation, lung transplantation, pancreas transplantation, and kidney–pancreas transplantation. We included all types of studies, including case reports, case series, and case-control studies that reported on SOT recipients who received CNIs as maintenance immunosuppressants and nirmatrelvir/ritonavir as a treatment for COVID-19.

## Results

### The studies characteristics

**Table 1** summarizes the characteristics of the study populations. A total of 13 articles, involving 84 SOT cases treated with NR, were included. Patient ages ranged from 14 to 79 years, with 50% (42/84) being men. The transplant types included 35 kidney transplants (41.6%), 22 heart transplants (26.2%), 21 lung transplants (25%), five liver transplants (6%), and one pancreas–kidney transplant (1.2%). Of the reported patients, 67 (79.8%) had been vaccinated, with 47 (70%) having received three doses or more. The time from transplant to COVID-19 ranged from 2 to 27.7 years.

**Table 1:** Study characteristics.

	Number of subject design	Age (years)	Gendermale/female	Organ transplant (%)	Time from txp to COVID-19 (years)	Vaccinated, ≥3 doses (%)	Maintenance immunosuppressant (%)
Wang et al. (12)	Four patients Case series	55 [40–70]	2/2	Kidney (100)	5 [2–9]	NA	Tac (100) MMF (100)
Salerno et al. (13)	25 patients Retrospective study Case series	57.7 (49.8–65.3)	14/11	Kidney (20) Liver (8) Lung (36) Heart (36)	3.6 (1.2–9.4)	23/25 16 (70)	Tac (84) CsA (16) mTORi (16)
Mecadon et al. (14)	Four patients Case series	43 [40–77]	3/1	Kidney (100)	4.8 [2–10]	NA	Tac (100) MMF (100) Pred (100)
Devresse et al. (15)	14 patients Single-center retrospective study	60 [33–79]	9/5	Kidney (100)	9.7 [0.8–27.7]	13/14 13 (100)	Tac (86) CsA (14) MMF (79) Pred (100)
Hedvat et al. (16)	28 patients Single-center retrospective study	57.6 (44.3–68.6)	11/17	Kidney (14) Liver (7) Lung (39) Heart (39)	3.9 (1.8–10.6)	25/28 15 (60)	NA
Guyon et al. (17)	One patient Case report	58	Woman	Liver	5	Yes 1	Tac MMF
Yanay et al. (18)	One patient Case report	23	Woman	Kidney	9	Yes 0	Tac Pred MMF
Prikis et al. (19)	One patient Case report	34	Man	Kidney	9	No	Tac Lef Pred
Rose et al. (20)	Two patients Case report	40	Man	Pancreas–kidney	7	Yes	Tac MMF
		58	Woman	Bilateral lung	6	NA	Tac Pred
Shah et al. (21)	One patient Case report	43	Man	Heart	NA	No	Tac
Lindauer et al. (22)	One patient Case report	41	Woman	Kidney	NA	NA	Tac
Young et al. (23)	One patient Case report	14	Girl	Kidney	2	Yes 1	Tac Pred
Stawiarski et al. (24)	One patient Case report	76	Woman	Heart	8	Yes 1	Tac MMF

Continuous variables are reported as median (IQR)/[ranges], and categorical variables are reported as n (%).

Tac, tacrolimus; CsA, cyclosporine; mTORi, mTOR inhibitor; MMF, mycophenolate acid; Pred, prednisone; Lef, leflunomide; NA, not available.

All studies reported the incidence of hospital admission or mortality as well as immunosuppressant drug levels and dose adjustments after NR treatment. Notably, the majority of patients received tacrolimus-based immunosuppressive therapy, which can affect NR administration and the clinical manifestations of SOTRs. Therefore, we focused on the use and monitoring of tacrolimus during NR therapy.

### The cases of holding CNIs/mTOR inhibitors during the nirmatrelvir/ritonavir treatment

A total of 75 cases (65 tacrolimus, six cyclosporines, three everolimus, and one sirolimus) from six studies were included in this analysis. Similar adjustment strategies were utilized for SOT patients receiving outpatient care, and all completed 5 days of nirmatrelvir/ritonavir treatment with slight variations in the timing of tacrolimus discontinuation and resumption of dose. **Table 2** summarizes the main findings.

**Table 2:** Studies of holding CNIs/mTOR inhibitors.

	ISDS dosing regular scheme	Days of restarting tacrolimus and % of baseline dose	Adverse reaction	Hospitalization (reason)	Outcome
Wang et al. (12)	Hold tacrolimus on the day of NR starting	10 [9–13] 67%–100%	One patient was diagnosed with AKI, due to self-discontinuing insulin.	0/4	All recovered from COVID-19 at the time of the last follow-up with no hospitalizations
Salerno et al. (13)	Hold tacrolimus or mTOR inhibitor or reduce cyclosporine dose to 20% of baseline daily dose	8 (7–9) 82%	No reported	4/25 (one for infectious diarrhea and three for symptoms related to COVID-19)	No patients were diagnosed with acute rejection or died within 30 days.
Hedvat et al. (16)	Hold tacrolimus or mTOR inhibitor or reduce cyclosporine dose to 20% of baseline daily dose	NA	One patient was diagnosed with stage one of AKI.	4/28 (any reasons)	No patients were diagnosed with acute rejection or died within 30 days.
Mecadon et al. (14)	Held tacrolimus after starting NR and resumed tacrolimus 24 h after completing therapy.	6 (night) 100%	No reported	0/3	No treatment complications appeared, and all did 6-month patient/allograft survival.
Devresse et al. (15)	Tacrolimus be held for 6 days and resumed on day 7. Reduce cyclosporine dose to 20% of baseline daily dose for 5 days. Rapamycin inhibitors dose to 12.5% of daily dose/48 h for 5 days.	7 100%	No reported	2/14 (acute pyelonephritis of the graft)	No patient died, and no viral pneumonia or acute rejection was diagnosed during the study period. Early relapses of COVID-19 symptoms occurred in two patients
Guyon et al. (17)	Hold tacrolimus and mycophenolate on the day of NR starting	6 (morning) 100%	Arthralgia, asthenia, and diarrhea; AKI; hyperkaliemia; and metabolic acidosis.	Yes (first kidney transplantation)	The concentrations of tacrolimus gradually decreased. Renal recovery occurred 2 months later.

Continuous variables are reported as median (IQR)/[ranges], and categorical variables are reported as n (%).

NR, nirmatrelvir/ritonavir; AKI, acute kidney injury.

In the study by Wang et al. (12), four kidney transplant recipients (KTRs) held tacrolimus on the day of NR initiation and resumed at 66%–100% of their baseline dose on days 9–13 (day 1 being the day of NR initiation). Mecadon et al. (14) described four KTRs who received NR for COVID-19; three of them held tacrolimus after starting NR and resumed tacrolimus 24 h after completing therapy. Similarly, Salerno et al. (13), Hedvat et al. (16), and Devresse et al. (15) adopted a similar dosing guideline: holding tacrolimus/mTOR inhibitors or reducing cyclosporine dose to 20% of the baseline daily dose on the day of NR initiation. In one liver transplant recipient who had recently undergone kidney transplantation, Guyon et al. (17) discontinued all immunosuppressants. Patients resumed taking tacrolimus either 12 h after the last dose of NR at 100% of the baseline dose (17), on day 7 (day 1 being the day of NR initiation) at 100% (15), or 3 days (2–5 days) after the completion of NR at 82% (interquartile range (IQR), 71–100) of their baseline dose (13).

In 85.3% (64/75) of patients, satisfactory trough concentrations of CNI were observed at the end of NR treatment; however, individual variability was high (12–14). Wang et al. (12) reported low or undetectable tacrolimus levels in two patients (2.7%, 2/75) on days 8–9 (day 1 being the day of NR initiation), while Salerno et al. (13) reported undetectable everolimus trough concentrations in two patients (2.7%, 2/75) on days 7 and 9. After resuming tacrolimus or cyclosporine for 3–4 days, the first assessment revealed a significant increase in CNI trough levels in 12% (9/75) of patients. Salerno et al. (13) reported one patient with a tacrolimus trough concentration of 24.6 ng/ml. Devresse et al. (15) reported a > 10-ng/ml increase in tacrolimus trough concentration for three patients and a > 350-ng/ml increase in cyclosporine for one patient. Mecadon et al. (14) reported that three patients experienced supratherapeutic levels (> 20 ng/ml), and Guyon et al. (17) reported a patient with a tacrolimus trough level of > 111 ng/ml.

During ISDs adjustment periods, most patients had a stable renal function. Two out of 75 patients (3%) experienced acute kidney injury (AKI), and one case was induced by tacrolimus nephrotoxicity (12, 17). No acute rejection or patient death occurred during the follow-up period of approximately 30 days. Out of the 75 patients, 13.3% (10/75) required hospitalization, with one case of infectious diarrhea, three cases related to COVID-19 symptoms (13), two cases of acute pyelonephritis (15), one case of general condition deterioration (17), and four cases of unknown reasons (16). Two patients experienced relapses of early SARS-CoV-2 infection, which may have been caused by infection with new variants, but both patients rapidly recovered within a few days of follow-up (15).

Notably, Hedvat et al. (16) reported that patients who received NR (*n* = 28) had a lower rate of 30-day hospitalization or death due to COVID-19 compared to those who received no specific treatment (*n* = 75) (10.7% vs. 30.7%, *p* = 0.043). Interestingly, there was no difference in the rate of hospitalization or death at 30 days among patients who received NR, regardless of whether they completed the COVID-19 vaccination series (≥ 3 doses) or not (0% vs. 25%, *p* = 0.113).

### The cases of keeping tacrolimus during nirmatrelvir/ritonavir treatment

Nine cases have been reported in which patients were prescribed NR as outpatients without holding tacrolimus but later required hospitalization due to serious adverse reactions or a surge in tacrolimus concentrations (14, 18–24). The clinical manifestations and course of treatment for these cases are presented in **Table 3**.

**Table 3:** Manifestations and outcomes of cases of keeping tacrolimus.

	NR regimen prior to hospitalization (doses)	Manifestations of toxicity	Treatment after admission	Outcomes (day 1 for admission)
Lindauer et al. (22)	5	Nausea, vomiting, and tremors; hyperkalemia; AKI	Hold Tac and NR	Tacrolimus and potassium returned to normal levels on day 7; serum creatinine returned to baseline on day 10
Rose et al. (20)	150/100 mg (4)	AKI; hepatic dysfunction	Reduce Tac dose to hold and administer oral rifampin 600 mg*3	Discharge on day 8 and later recovered from his COVID-19 infection
	300/100 mg (6)	Abdominal pain, nausea and vomiting enteritis, and somnolence; AKI	Intravenous fluids, dexamethasone, remdesivir, vancomycin, and ceftriaxone for pneumonia. Hold Tac and administer oral rifampin 300 mg*4	Abdominal pain and enteritis, attributed to tacrolimus toxicity, had completely resolved on day 8. Later recovered from COVID-19.
Mecadon et al. (14)	10	Weakness, confusion, and AKI.	Hold Tac	Resolution of confusion and AKI
Yanay et al. (18)	300/100 mg (3)	No clinical manifestations of a tacrolimus overdose	Gradually reduce Tac dose to hold	Clinical condition improved, creatinine level returned to the baseline level, and discharged on day 7
Young et al. (23)	300/100 mg (2)	No neurologic symptoms, no electrolyte derangements.	Hold Tac and NR, received monoclonal antibody (bebtelovimab) infusion then adjust Tac dose	COVID-19 symptoms are completely resolved.
Prikis et al. (19)	150/100 mg (5)	Nausea, vomiting, AKI	Hold Tac and NR	Symptom resolved completely 7 days after stopping NR
Shah et al. (21)	NA	Worsening cough, dyspnea, hemoptysis, hyperkalemia, normal anion-gap metabolic acidosis, AKI, chronic pancytopenia	Oxygen inhalation inhaled tranexamic acid Hold Tac and treated with phenytoin	Tac return to normal levels and discharged home on room air within 2 weeks of initial admission.
Stawiarski et al. (24)	300/100 mg (4)	AKI	Hold Tac and NR, received monoclonal antibody therapy with 175 mg of bebtelovimab, no other special treatment	Resolution of acute kidney injury on day 7.

NR, nirmatrelvir/ritonavir; Tac, tacrolimus; AKI, acute kidney injury.

Among these cases, only one patient (11.1%) completed the full NR treatment course, while the others either were admitted to the hospital for adverse reactions or notified their transplant care team and were promptly instructed to stop the medication while monitoring tacrolimus concentrations. All the cases reported supratherapeutic tacrolimus levels (> 30 ng/ml), and only two cases showed no significant clinical manifestations. Common adverse reactions reported during treatment with NR included AKI (78%, 7/9); gastrointestinal symptoms such as nausea, vomiting, and abdominal pain (33%, 3/9); neurologic symptoms such as tremors, confusion, and somnolence (33%, 3/9); and electrolyte disturbances (33%, 3/9). Other adverse events, such as hepatic dysfunction and pancytopenia, were less common. Most of these adverse reactions were likely due to tacrolimus toxicity. The median time for the tacrolimus concentration to return to baseline levels after holding NR was 8 (IQR, 7–12) days. Notably, in two case reports (20, 21), rifampin and phenytoin were used to accelerate tacrolimus metabolism, resulting in a rapid decline in therapeutic levels.

As to the concurrent management of steroids and antimetabolite drugs, Devresse et al. (15) kept the steroid dose constant; Yanay et al. (18) doubled the prednisone dose while maintaining mycophenolic acid; and Rose et al. (20) also maintained mycophenolic acid. There was no report on the effect of this adjustment.

## Discussion

In this study, we conducted a thorough review of all available evidence regarding SOTRs who have been infected by SARS-CoV-2 and have received nirmatrelvir/ritonavir in conjunction with immunosuppressants. Our focus was on the potential drug interactions and the adjustment of immunosuppressant dosages during treatment.

Due to their dependence on CYP3A metabolism and P-glycoprotein-mediated transport, CNIs pose a significant risk for drug interactions when co-administered with ritonavir, a well-known irreversible potent inhibitor of CYP3A (25). Ritonavir has been shown to significantly increase the exposure of tacrolimus by up to 40-fold (26). To reduce or avoid this drug interaction, two key phases must be considered: the initiation of NR and empirical adjustments to baseline CNI dosing, and the reintroduction or dose adjustment of CNIs following the completion of NR treatment.

We compared patients who stopped taking tacrolimus during NR treatment with those who continued or reduced their dosage. As expected, the former group had better clinical outcomes, with lower rates of hospitalization (13.3% vs. 89.9%) and tacrolimus toxicity (4% vs. 78%) during the follow-up period. Although some patients in the tacrolimus-holding group had low or undetectable tacrolimus concentrations during the 5-day treatment period, no cases of acute rejection were reported, and tacrolimus concentrations remained relatively stable. Therefore, holding tacrolimus at the start of NR treatment appears to be a simple and safe strategy to avoid overexposure. A study has suggested reducing tacrolimus dosage by approximately 0.5 mg once weekly to prevent toxicity when co-administered with ritonavir-boosted antiretroviral therapy (27), and another research also considered a tacrolimus dose of less than 1 mg/week may be sufficient to maintain adequate blood tacrolimus concentrations in patients on Kaletra (lopinavir and ritonavir) (28), which were worth considering. Additionally, for patients with high immunologic risk factors, such as those in the early posttransplant period or with a high risk of rejection, one study recommended administering a single 1/8 of the daily tacrolimus dosage on the first day of NR treatment and then no further doses for the rest of the treatment period. This approach can maintain comparable total exposure during the 5-day antiviral treatment while limiting overexposure and preventing rejection (29).

According to our analysis of the included studies, there was no consensus on the optimal timing and dosing of tacrolimus re-initiation. Some studies, such as those by Salerno et al. (13) and Wang et al. (12), implemented an individualized resumption strategy for their patients, administering the adjusted tacrolimus dose based on monitoring levels, with satisfactory results. We also observed that in some cases, supratherapeutic levels of tacrolimus persisted for up to 10 days after partial or full dose resumption, despite low tacrolimus concentrations, due to a longer CYP3A4 inhibition induced by NR administration (13). A simulation study of lopinavir/ritonavir treatment cessation showed a gradual decrease in drug transport and CYP3A inhibition, with 50% recovery of metabolism after 24 hours and 75% after 48 hours (30). Therefore, Lemaitre et al. proposed reintroducing tacrolimus at 50% of the daily dose on Day 6 (Day 1 for the initiation of NR) and increasing it to 75% on day 7 before resuming the usual daily dose on day 8 (29). Along with monitoring whole blood tacrolimus concentrations, qualified institutions can measure nirmatrelvir and ritonavir plasma concentrations to better determine metabolic status and the appropriate timing of tacrolimus administration. A recent study has reported a simple and rapid UPLC-MS/MS method for the quantitative determination of NR in human plasma, which may improve drug guidance (17).

Our literature search yielded limited evidence to support specific adjustment schemes for other ISDs. **Table 4** provides a summary of the available guidance and published recommendations on the adjustment strategy of immunosuppressive drugs when co-administered with nirmatrelvir/ritonavir (9, 10, 26, 31, 32). Based on current experience, cyclosporine should be reduced to 20% of the baseline daily dose (DD) upon initiation of NR and gradually reintroduced from day 6, following a similar approach to tacrolimus resumption. As for mTOR inhibitors, it is recommended that they be stopped during the administration of NR or administered at 1/8 of the usual DD on days 1, 3, and 5, and the usual DD be reintroduced on day 7. Our included studies have reported little evidence about mycophenolic acid and steroid management. Current guidelines suggest that mycophenolic acid and steroid doses may remain unchanged, as a weak interaction is expected (26, 32). These recommendations are primarily theoretical, and individualized drug concentration monitoring is necessary due to the inhibitory effect of ritonavir on various immunosuppressive agents. Further clinical studies are needed to explore the most appropriate strategy for these drugs with NR in transplant patients.

**Table 4:** A summary of adjustment strategies of immunosuppressive drugs.

	Tacrolimus		Cyclosporine	mTOR inhibitors	Mycophenolic acid	Corticosteroids
EUA OF FDA (10) December 2021	Avoid the use of Paxlovid when close monitoring of immunosuppressant concentrations is not feasible. If co-administered, dose adjustment of the immunosuppressant is recommended.			Avoid concomitant use of everolimus and sirolimus and Paxlovid.	NA	NA
Fishbane et al. (9) January 2022	Recommendation: reduce or hold CNI and mTORi dose following the first dose of Paxlovid; base subsequent dosing on trough levels; if trough below target goal, administer a single dose of CNI; resume normal CNI or mTORi dose once the treatment course of Paxlovid finished.				NA	NA
Lange et al. (31) January 2022	Hold Tac on days 1–5 of NR treatment If feasible, measure a Tac level on day 3 to assess the need for a one-time supplementary Tac dose.		Recommend an empiric dose reduction for daily dose by 80%	NA	NA	NA
	The CNI level should be assessed as soon as possible upon N/R completion. Resumption of Tac or dose increases of CsA should begin once drug levels approach the therapeutic target.					
SFPT (26) April 2022	High immunological risk^*^: administer 1/8th of the usual DD on day 1, then stop. Administer 1/2nd of the DD on day 6 then 3/4 on day 7 and restart the usual DD on day 8.	Low immunological risk: Discontinue Tac 12 h before initiation of N/R. Restart Tac at the usual DD 24 h after the last antiviral dose.	Administer 1/5th of the usual DD every day of nirmatrelvir/ritonavir treatment. Administer 1/2nd of the DD on day 6 then 3/4 on day 7 and restart the usual DD on day 8.	Administer 1/8th of the usual DD on days 1, 3, and 5. Usual DD can be restarted on day 7.	Maintain drug dosage.	Maintain drug dosage. (If needed, a 1/3 dosage decrease can also be proposed.)
Lemaitre et al. (32) August 2022	Hold Tac 12 h before initiation of N/R. OR: administer 1/8th of the usual daily dose on day 1 and stop. Reintroduced usual DD on day 7/8.		Reduce to 20% of the initial dose on day 1. Progressively reintroduced from day 6.	Hold mTOR inhibitor for 12 h before initiation of N/R. OR: administer 1/8th of the usual daily dose on days 1, 3, and 5. Reintroduced the usual DD on day 7.	No need to adjust drug dosage.	No need to adjust drug dosage.

All guidelines and suggestions are recommendations, and dosage with treatment individualization using therapeutic drug monitoring if possible.

^*^Patients in the early posttransplant period or those with a high risk of rejection.

CNI, calcineurin inhibitors; NR, nirmatrelvir/ritonavir; Tac, tacrolimus; CsA, cyclosporine; mTORi, mTOR inhibitors; DD, daily dose; NA, not available.

However, there are special circumstances where transplant patients may first seek medical attention from primary care providers who may not be aware of the potential drug interactions between ritonavir and CNIs or mTOR inhibitors during the COVID-19 pandemic. In SOT patients who took Paxlovid without adjusting their CNIs, especially tacrolimus, most experienced a significant increase in tacrolimus levels and severe complications such as AKI, neurotoxicity, and gastrointestinal symptoms. Fortunately, most transplant patients recovered after prompt drug withdrawal and hospital care. Notably, two studies have reported that phenytoin and rifampicin can be used to accelerate tacrolimus metabolism and excretion, resulting in a significant reduction in tacrolimus concentration (20, 21). Phenytoin and rifampin are potent inducers of CYP3A4 and P-glycoprotein, which can quickly reduce tacrolimus levels as antidotes (33, 34). Therefore, clinicians should consider these medications when transplant patients require hospitalization due to a severe increase in tacrolimus concentration caused by Paxlovid administration.

Similar to the findings in the general population, NR has been shown to be effective in preventing COVID-19-related hospitalization and death in SOTRs, as demonstrated by Hedvat et al. (16). Evidence has suggested that the BA.2 subvariant of Omicron is substantially more infectious than previous Omicron variants. A case series conducted by Devresse et al. (15) demonstrated the effectiveness of NR in SOTRs infected with the Omicron BA.2 variant. None of the patients required hospitalization for viral pneumonia, and the viral load of all patients had decreased substantially by day 7. These findings suggest that during the pandemic of Omicron variants, NR could be an effective treatment option for COVID-19 in SOTRs, provided that immunosuppressive regimens are properly managed.

Moreover, it is important to note that the inhibition of CYP3A by nirmatrelvir/ritonavir can also lead to potential drug interactions with other medications commonly prescribed to transplant recipients for different comorbidities. These medications include HMG-CoA reductase inhibitors (statins), azole antifungals, calcium channel blockers, and anticoagulants like warfarin, which may need to be either held or adjusted during the administration of nirmatrelvir/ritonavir therapy (26).

It is also worth mentioning that molnupiravir is an oral ribonucleoside antiviral agent that is authorized for emergency use in the outpatient treatment of mild-to-moderate COVID-19 patients within five days of symptom onset and that it does not exhibit the drug interactions observed with NR. But the MOVe­OUT phase 3 trial has demonstrated a roughly 30% relative risk reduction of hospitalization or death at day 29 for patients who received molnupiravir versus placebo (35), which indicates that NR is more effective than molnupiravir. Transplant patients may experience rapid disease changes, and guidelines recommend Molnupinavir as a second-line agent (36). Therefore, most transplant physicians choose NR as their first choice.

This review has several limitations. Firstly, all studies included in this review were either case reports or case series, with only one study having a control group. Additionally, the sample sizes of the studies were limited. Secondly, most of the studies were retrospective, and the follow-up period was less than 30 days. Therefore, it is not possible to conclude that withholding CNIs is safe in terms of immunological risk and the occurrence of rejection. Thirdly, most patients in the studies received tacrolimus, with a few cases receiving cyclosporine and mTOR inhibitors. Therefore, it is difficult to draw an informative conclusion regarding the most appropriate strategy for these drugs when used with NR in transplant patients. Further clinical studies are needed to better understand the optimal use of NR in this population.

## Conclusion

In conclusion, our analysis indicates that nirmatrelvir/ritonavir can be a promising therapeutic option for SOT recipients with COVID-19. However, careful drug adjustments and monitoring are necessary to minimize the risk of adverse effects and harm to the allograft. It is, therefore, crucial to actively monitor drug concentration levels to prevent toxicity and adjust immunosuppressive agents accordingly. Collaborative management of COVID-19 in transplant patients involving primary care providers, pharmacists, and transplant teams is essential for optimizing patient outcomes.

## Author contributions

YT made the literature search and wrote the manuscript; YL collected the data; TS designed the study and revised the manuscript. All authors reviewed the manuscript. All authors contributed to the article and approved the submitted version.
